# Supplementary material for: Characterization and Analysis of the Complete Mitochondrial Genome of Platycrater arguta
Source: Curr Issues Mol Biol. 2025 Jul 5;47(7):521. doi: 10.3390/cimb47070521 (PMC12293517; doi:10.3390/cimb47070521)
Supplement: Supplementary file 1 [file cimb-47-00521-s001.zip › cimb-3692170-supplementary.pdf]

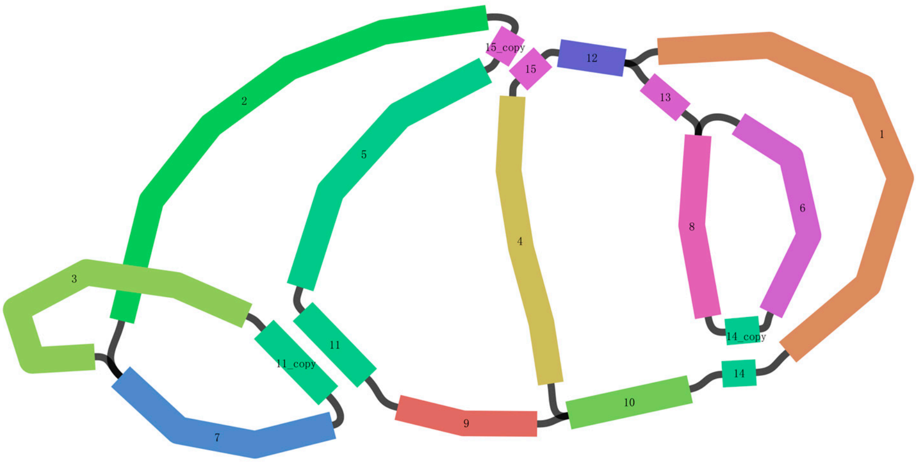

Figure S1. Graph model after solved the 3 repeats.

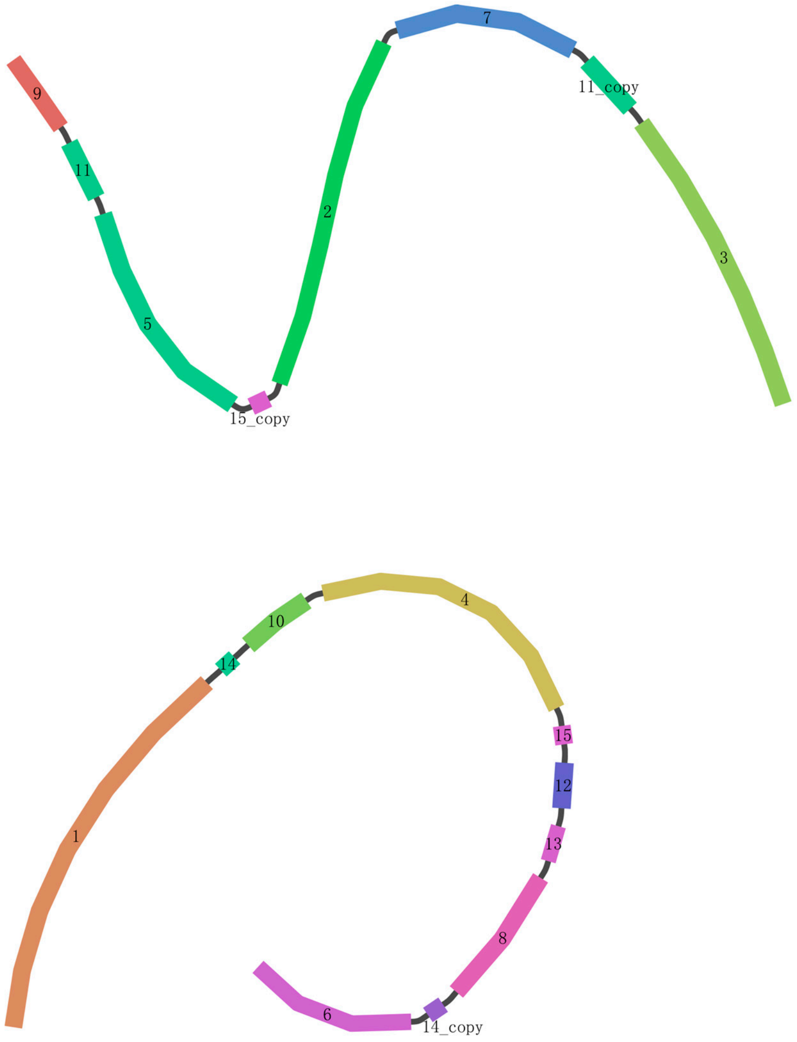

Figure S2. mtDNA processed into 2 molecules for convenience of analysis.

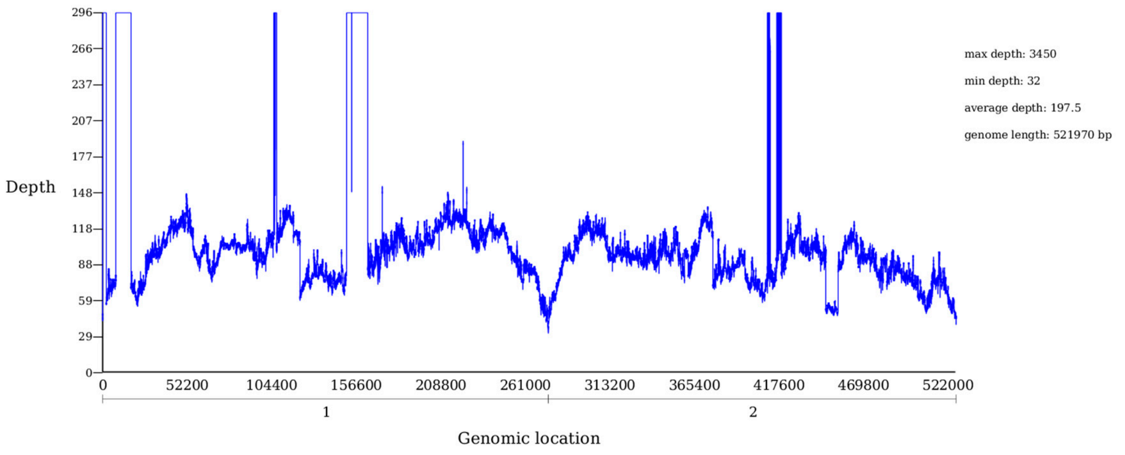

Figure S3. The coverage plot of the third-generation sequencing data.

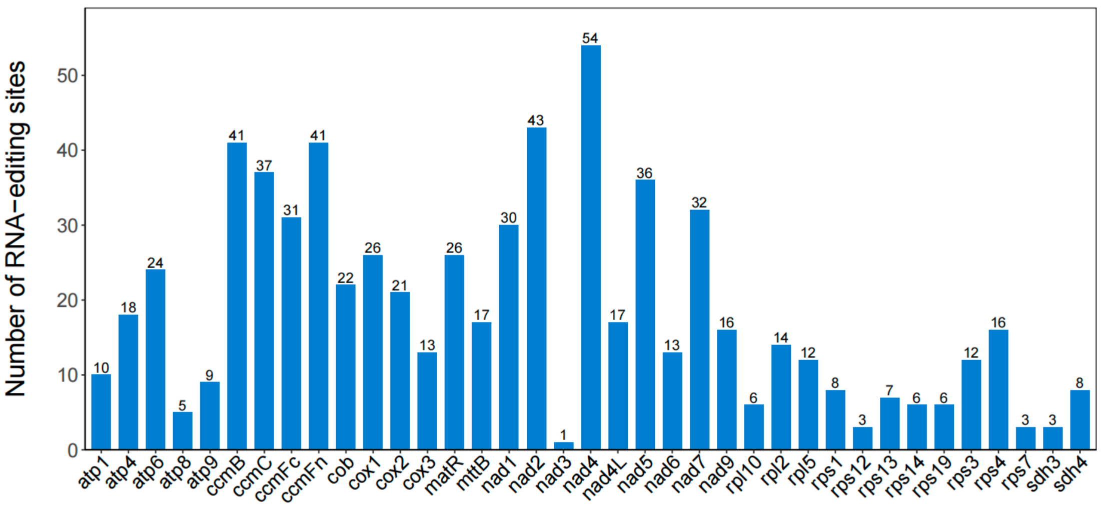

Figure S4. The distribution of RNA-editing sites of each PCG in the *P. arguta* mt genome.

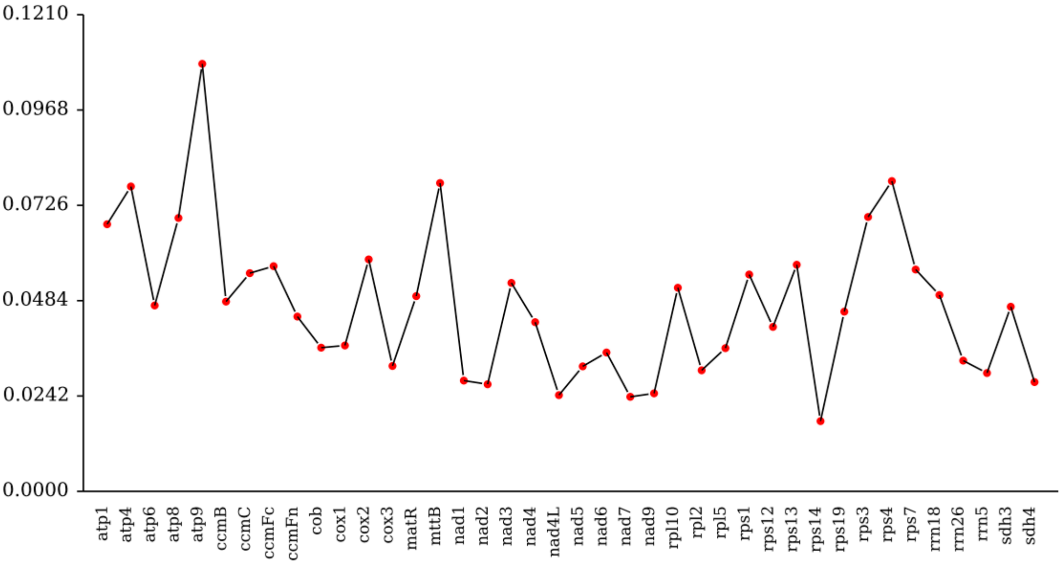

Figure S5. Line graph of 37 PCGs Pi in *P. arguta* mitogenome.

Table S1. Third-generation sequencing data of the *P. arguta* mt genome.

Number of reads	Number of bases	Mean read length (bp)	N50 read length (bp)
591417	10396477834	17578	17594

Table S2. Basic information of chromosomes and path selection for each node based on HiFi data.

Contig	Type	Path	Length	GC content
1	linear	9-11-5-15-2-7-11-3	272,565 bp	45.66%
2	linear	1-14-10-4-15-12-13-8-14-6	249,405 bp	45.58%
Total			521,970 bp	45.62%

Table S3. Gene profile and organization of the *P. arguta* mitogenome.

Group of genes	Gene name	Length	Start codon	Stop codon	Amino acid
ATP synthase	*atp1*	1530	ATG	TGA	510
	atp4	579	ATG	TAA	193
	atp6	768	ATG	CAA(TAA)	256
	atp8	480	ATG	TAA	160
	atp9	225	ATG	CGA(TGA)	75
Cytochrome c biogenesis	*ccmB*	621	ATG	TGA	207
	ccmC	720	ATG	TAG	240
	ccmFc[a]	1317	ATG	CGA(TGA)	439
	ccmFn	1734	ATG	TGA	578
Ubichinol cytochrome c reductase	*cob*	1182	ATG	TGA	394
Cytochrome c oxidase	*cox1*	1584	ACG(ATG)	TAA	528
	cox2[a]	792	ATG	TAA	264
	cox3	798	ATG	TGA	266
Maturase	*matR*	1968	ATG	TAG	656
Transport membrance protein	*mttB*	348	ATG	TAG	116
NADH dehydrogenase	*nad1*[aa]	978	ATG	TAA	326
	nad2[aa]	1467	ATG	TAA	489
	nad3	357	ATG	TAA	119
	nad4[aaa]	1488	ATG	TGA	496
	nad4L	303	ACG(ATG)	TAA	101
	nad5[aaa]	2013	ATG	TAA	671
	nad6	618	ATG	TAA	206
	nad7[aaaa]	1185	ATG	TAG	395
	nad9	573	ATG	TAA	191

Ribosomal proteins (LSU)	*rpl10*	489	ATG	TAA	163
	rpl2	1014	ATG	TAA	338
	rpl5	564	ATG	TAA	188
Ribosomal proteins (SSU)	*rps1*	606	ATG	TAA	202
	rps12	378	ATG	TGA	126
	rps13	351	ATG	TGA	117
	rps14	303	ATG	TAG	101
	rps19	291	ATG	TAA	97
	rps3[a]	1692	ATG	TAG	564
	rps4	831	ATG	TAA	277
	rps7	447	ATG	TAA	149
Succinate dehydrogenase	*sdh3*	327	ATG	TGA	109
	sdh4	387	ATG	CGA(TGA)	129
Ribosomal RNAs	*rrn18*	1935			
	rrn26	3598			
	rrn5	121			
Transfer RNAs	*trnA-TGC*	65			
	trnC-GCA	71			
	trnD-GTC	74			
	trnE-TTC	72			
	trnF-GAA	73			
	trnF-GAA	74			
	trnG-GCC	72			
	trnH-GTG	74			
	trnI-GAT	72			
	trnK-TTT	73			
	trnK-TTT	73			
	trnM-CAT	74			
	trnM-CAT	73			
	trnM-CAT	74			
	trnM-CAT	73			
	trnN-GTT	72			
	trnP-TGG	75			
	trnP-TGG	74			
	trnQ-TTG	72			
	trnR-ACG	74			
	trnS-GCT	88			
	trnS-TGA	87			
	trnT-TGT	72			
	trnT-TGT	73			
	trnV-GAC	72			
	trnW-CCA	74			
	trnY-GTA	83			

[a] Intron number

Table S4. RSCU analysis of each amino acid in the *P. arguta* mt genome.

AminoAcid	Symbol	Codon	No.	RSCU
*	Ter	UAA	19	1.5405
*	Ter	UAG	6	0.4865
*	Ter	UGA	12	0.973
A	Ala	GCA	164	0.988
A	Ala	GCC	156	0.9398
A	Ala	GCG	78	0.4699
A	Ala	GCU	266	1.6024
C	Cys	UGC	54	0.766
C	Cys	UGU	87	1.234
D	Asp	GAC	105	0.625
D	Asp	GAU	231	1.375
E	Glu	GAA	292	1.3303
E	Glu	GAG	147	0.6697
F	Phe	UUC	300	0.9259
F	Phe	UUU	348	1.0741
G	Gly	GGA	265	1.456
G	Gly	GGC	96	0.5275
G	Gly	GGG	129	0.7088
G	Gly	GGU	238	1.3077
H	His	CAC	63	0.4884
H	His	CAU	195	1.5116
I	Ile	AUA	216	0.81
I	Ile	AUC	231	0.8662
I	Ile	AUU	353	1.3237
K	Lys	AAA	261	1.1626
K	Lys	AAG	188	0.8374
L	Leu	CUA	161	0.9028
L	Leu	CUC	117	0.6561
L	Leu	CUG	97	0.5439
L	Leu	CUU	231	1.2953
L	Leu	UUA	245	1.3738
L	Leu	UUG	219	1.228
M	Met	AUG	288	1
N	Asn	AAC	109	0.6626
N	Asn	AAU	220	1.3374
P	Pro	CCA	178	1.1769
P	Pro	CCC	114	0.7537
P	Pro	CCG	95	0.6281
P	Pro	CCU	218	1.4413
Q	Gln	CAA	218	1.5087

Q	Gln	CAG	71	0.4913
R	Arg	AGA	170	1.4246
R	Arg	AGG	88	0.7374
R	Arg	CGA	160	1.3408
R	Arg	CGC	69	0.5782
R	Arg	CGG	85	0.7123
R	Arg	CGU	144	1.2067
S	Ser	AGC	104	0.6316
S	Ser	AGU	165	1.002
S	Ser	UCA	186	1.1296
S	Ser	UCC	164	0.996
S	Ser	UCG	138	0.8381
S	Ser	UCU	231	1.4028
T	Thr	ACA	122	0.9208
T	Thr	ACC	144	1.0868
T	Thr	ACG	76	0.5736
T	Thr	ACU	188	1.4189
V	Val	GUA	194	1.1902
V	Val	GUC	120	0.7362
V	Val	GUG	145	0.8896
V	Val	GUU	193	1.184
W	Trp	UGG	154	1
Y	Tyr	UAC	75	0.4762
Y	Tyr	UAU	240	1.5238

Table S5. The tandem repeats in *P. arguta* mitogenome.

NO	Contig	Size	Copy	Repeat sequence	Percent Matches	Start	End
1	contig1	18	1.9	ATCTCGCATCTAGATCTGT	88	5806	5841
2	contig1	15	2.3	TTCACTCCTACGCTC	89	48530	48563
3	contig1	21	2	TTCCTTTCAAGCTACTACCAA	82	51572	51615
4	contig1	19	2	TAAGACTAGAGTAGCTGGT	94	58139	58176
5	contig1	15	2	TGAATGCAATAACTT	93	87647	87676
6	contig1	16	2	TCTCATTAAAGACTCA	93	127536	127567
7	contig1	25	2	CTTTCAAAGTAGTCCCTAAACTCGCG	92	127780	127829
8	contig1	16	2	CTTCATACACATATAC	100	152199	152230
9	contig1	12	2.5	CTGACTTTCCTA	94	179933	179962
10	contig1	20	3	ATTGCTATTGATTTCTTCGCT	80	186989	187052
11	contig1	16	2.1	TTGCTTGCTACTTAGT	88	191537	191570
12	contig1	18	1.9	AAGGTTATCAAGTACAAG	94	195622	195656
13	contig1	32	2.4	TTACCTCTTACCTAGGCCCTGTTCGCTTTAC	75	208677	208753
14	contig1	19	2.1	AAAATAATTTTATTTATT	86	258421	258460
15	contig1	13	2	CTTAAAAAGAGCT	100	264788	264813
16	contig2	18	2.4	GACTATGAAACAGATCGC	81	29983	30025
17	contig2	16	2	ACTAACCACTTTAGCA	93	39403	39434
18	contig2	16	2	GTAGTGTCTATCCCTG	93	43218	43249
19	contig2	18	2.2	CAGAGAAAGTAGTCCAAC	90	53152	53191
20	contig2	11	2.5	TATAGCATACC	100	76251	76277
21	contig2	18	2	TCTTGTCGATACCTACTAG	89	152964	153000
22	contig2	17	1.9	AAGATACCCTAAATTTG	93	180137	180169
23	contig2	8	7.1	GCCTTAAA	100	211169	211225
24	contig2	5	15.2	GAAGG	100	211231	211306
25	contig2	15	2	CCTACTATACAATAC	93	233750	233779
26	contig2	39	2	TTCACTCATGATCTGGCCTGGTCGACCCAATCATGATAT	97	234674	234752

Table S6. Dispersed repeats analysis in the *P. arguta* mt genome.

#Contig1	Contig2	type	length	similarity	start1	end1	start2	end2	evalue
contig1	contig1	F	12915	100	240423	253337	68741	81655	0
contig1	contig1	F	202	99.505	168614	168815	139882	140083	1.56e-100
contig1	contig1	P	887	73.506	235159	236017	9608	10471	1.25e-76
contig1	contig1	P	145	100	123395	123539	32619	32763	1.62e-70
contig1	contig1	F	142	96.479	254412	254553	131109	131250	1.65e-60
contig1	contig1	P	99	100	131264	131362	26377	26475	6.05e-45
contig1	contig1	P	257	78.988	234518	234772	10498	10754	3.64e-42
contig1	contig1	F	90	100	231816	231905	40817	40906	6.10e-40
contig1	contig1	P	90	100	127245	127334	75897	75986	6.10e-40
contig1	contig1	P	90	100	247579	247668	127245	127334	6.10e-40
contig1	contig1	F	81	97.531	109581	109661	95089	95169	1.33e-31
contig1	contig1	P	96	92.708	257992	258087	233855	233945	6.18e-30
contig1	contig1	F	77	96.104	190615	190691	13493	13569	1.03e-27
contig1	contig1	F	70	97.143	271344	271413	87925	87994	1.73e-25
contig1	contig1	F	68	95.588	233909	233976	22480	22547	1.04e-22
contig1	contig1	F	75	92	198226	198300	197260	197332	4.85e-21
contig1	contig1	P	109	84.404	55306	55407	21799	21906	6.27e-20
contig1	contig1	P	182	78.022	21799	21973	55235	55407	6.27e-20
contig1	contig1	F	62	95.161	109295	109356	25520	25581	2.26e-19
contig1	contig1	P	54	98.148	215559	215612	205350	205403	2.92e-18
contig1	contig1	P	72	90.278	213087	213158	156589	156659	1.05e-17

contig1	contig1	F	49	100	269037	269085	35829	35877	3.77e-17
contig1	contig1	F	64	92.188	239417	239479	37445	37504	1.76e-15
contig1	contig1	P	72	88.889	192089	192160	118210	118278	1.76e-15
contig1	contig1	F	67	89.552	260411	260477	131402	131468	1.76e-15
contig1	contig1	F	68	89.706	221626	221692	197756	197823	1.76e-15
contig1	contig1	P	54	94.444	188995	189048	32744	32797	6.32e-15
contig1	contig1	P	48	97.917	130840	130887	37700	37747	6.32e-15
contig1	contig1	F	45	100	258291	258335	129030	129074	6.32e-15
contig1	contig1	P	57	92.982	32744	32800	188992	189048	6.32e-15
contig1	contig1	F	56	92.857	239207	239261	111205	111260	8.17e-14
contig1	contig1	F	47	97.872	267033	267079	190931	190976	8.17e-14
contig1	contig1	P	77	85.714	55432	55505	148	224	2.94e-13
contig1	contig1	P	80	85	148	227	55429	55505	2.94e-13
contig1	contig1	F	47	95.745	110862	110908	109891	109937	1.06e-12
contig1	contig1	P	151	76.821	132901	133046	132898	133043	1.06e-12
contig1	contig1	P	47	95.745	181188	181234	132903	132949	1.06e-12
contig1	contig1	F	51	92.157	268270	268320	234937	234987	1.37e-11
contig1	contig1	F	41	97.561	59828	59868	57925	57965	4.92e-11
contig1	contig1	F	53	90.566	215017	215069	233061	233113	4.92e-11
contig1	contig1	P	37	100	184663	184699	8092	8128	1.77e-10
contig1	contig1	P	61	86.885	236203	236263	9403	9463	1.77e-10
contig1	contig1	P	37	100	257992	258028	22480	22516	1.77e-10
contig1	contig1	P	44	95.455	268523	268565	268280	268323	1.77e-10
contig1	contig1	F	39	97.436	22177	22181	38767	38805	6.36e-10

					6	4			
contig1	contig1	P	93	80.645	120777	120868	55312	55399	6.36e-10
contig1	contig1	F	36	100	236283	236318	122335	122370	6.36e-10
contig1	contig1	P	59	88.136	238389	238446	151886	151941	6.36e-10
contig1	contig1	F	39	97.436	238329	238367	234238	234276	6.36e-10
contig1	contig1	P	71	84.507	151886	151953	238377	238446	6.36e-10
contig1	contig1	F	168	75.595	120777	120929	21807	21969	2.29e-09
contig1	contig1	F	71	84.507	140750	140819	25516	25582	2.29e-09
contig1	contig1	P	35	100	242934	242968	70895	70929	2.29e-09
contig1	contig1	P	35	100	71252	71286	70895	70929	2.29e-09
contig1	contig1	P	35	100	242577	242611	71252	71286	2.29e-09
contig1	contig1	P	35	100	242934	242968	242577	242611	2.29e-09
contig1	contig1	P	34	100	190910	190943	14112	14145	8.23e-09
contig1	contig1	F	70	84.286	215036	215100	197782	197851	8.23e-09
contig1	contig1	P	51	88.235	234350	234400	10825	10875	2.96e-08
contig1	contig1	P	37	97.297	130050	130086	22475	22510	2.96e-08
contig1	contig1	F	49	89.796	267003	267051	110108	110155	2.96e-08
contig1	contig1	P	44	90.909	174722	174765	7860	7903	1.06e-07
contig1	contig1	P	35	97.143	178312	178346	25207	25241	1.06e-07
contig1	contig1	F	32	100	238561	238592	68599	68630	1.06e-07
contig1	contig1	P	35	97.143	268639	268673	171736	171770	1.06e-07
contig1	contig1	F	42	92.857	174516	174557	172884	172924	1.06e-07
contig1	contig1	P	41	92.683	265060	265099	73820	73860	3.83e-07

contig1	contig1	F	66	83.333	178311	178373	132875	132940	3.83e-07
contig1	contig1	P	35	97.143	261717	261750	134532	134566	3.83e-07
contig1	contig1	P	50	88	239403	239451	137183	137231	3.83e-07
contig1	contig1	F	34	97.059	233134	233167	221181	221214	3.83e-07
contig1	contig1	P	38	94.737	236914	236951	236486	236522	3.83e-07
contig1	contig1	P	40	92.5	137192	137231	239403	239442	3.83e-07
contig1	contig1	P	41	92.683	265060	265099	245502	245542	3.83e-07
contig1	contig1	P	33	96.97	196335	196367	3465	3497	1.38e-06
contig1	contig1	F	85	80	45274	45353	44942	45024	1.38e-06
contig1	contig1	F	34	97.059	215170	215203	93680	93712	1.38e-06
contig1	contig1	F	65	83.077	140754	140818	109295	109356	1.38e-06
contig1	contig1	F	36	94.444	181199	181234	133006	133041	1.38e-06
contig1	contig1	P	52	86.538	164192	164243	137195	137244	1.38e-06
contig1	contig1	P	56	85.714	163464	163517	163464	163517	1.38e-06
contig1	contig1	P	36	94.444	3465	3500	196332	196367	1.38e-06
contig1	contig1	P	39	92.308	268527	268565	234947	234985	1.38e-06
contig1	contig1	F	29	100	255356	255384	3530	3558	4.95e-06
contig1	contig1	F	44	88.636	109011	109054	24694	24737	4.95e-06
contig1	contig1	F	29	100	70366	70394	69600	69628	4.95e-06
contig1	contig1	F	29	100	242048	242076	69600	69628	4.95e-06
contig1	contig1	F	29	100	241282	241310	70366	70394	4.95e-06
contig1	contig1	F	40	92.5	257998	258034	130050	130089	4.95e-06
contig1	contig1	P	41	90.244	196332	196372	132803	132843	4.95e-06

contig1	contig1	F	32	96.875	174734	174765	135574	135605	4.95e-06
contig1	contig1	F	36	94.444	268290	268325	171697	171730	4.95e-06
contig1	contig1	F	29	100	242048	242076	241282	241310	4.95e-06
contig1	contig2	P	623	100	190743	191365	159902	160524	0
contig1	contig2	F	329	100	64397	64725	18841	19169	8.43e-173
contig1	contig2	F	302	99.669	266874	267175	199871	200172	4.00e-156
contig1	contig2	F	314	95.86	38491	38803	66949	67262	3.16e-142
contig1	contig2	F	239	91.632	260444	260673	72299	72537	1.23e-86
contig1	contig2	P	184	97.826	52808	52991	65617	65800	1.59e-85
contig1	contig2	F	158	100	32674	32831	116244	116401	9.64e-78
contig1	contig2	P	156	99.359	37438	37593	31571	31726	5.80e-75
contig1	contig2	P	151	99.338	256831	256981	239915	240065	3.49e-72
contig1	contig2	P	153	98.039	34372	34524	227320	227472	5.84e-70
contig1	contig2	P	359	79.387	15651	16003	28855	29211	7.61e-64
contig1	contig2	F	151	96.026	47022	47170	93696	93846	2.74e-63
contig1	contig2	F	131	100	37583	37713	66307	66437	9.85e-63
contig1	contig2	F	134	99.254	24102	24235	185141	185274	9.85e-63
contig1	contig2	F	124	99.194	131232	131355	93585	93708	3.57e-57
contig1	contig2	P	123	99.187	221088	221209	100471	100593	4.61e-56
contig1	contig2	P	116	100	50759	50874	24100	24215	2.15e-54
contig1	contig2	P	463	75.81	14923	15369	29477	29920	1.67e-50
contig1	contig2	F	109	100	131011	131119	133376	133484	1.67e-50
contig1	contig2	P	293	79.863	101466	101747	41095	41378	7.78e-49
contig1	contig2	F	128	94.531	257670	257797	68983	69109	7.78e-49
contig1	contig2	F	126	95.238	198035	198159	143680	143802	7.78e-49
contig1	contig2	F	121	94.215	54647	54761	10251	10263	7.83e-44

							3	3	
contig1	contig2	F	104	96.154	34422	34525	7476	7579	4.71e-41
contig1	contig2	P	96	98.958	267190	267284	72922	73017	4.71e-41
contig1	contig2	P	92	100	26384	26475	93617	93708	4.71e-41
contig1	contig2	P	90	100	123395	123484	116244	116333	6.10e-40
contig1	contig2	F	113	92.92	197264	197376	240487	240595	2.84e-38
contig1	contig2	P	76	100	26401	26476	72209	72284	3.70e-32
contig1	contig2	F	75	100	131264	131338	72210	72284	1.33e-31
contig1	contig2	P	85	96.471	110063	110145	85531	85615	1.33e-31
contig1	contig2	F	103	91.262	256043	256145	213347	213448	1.33e-31
contig1	contig2	P	81	97.531	26489	26569	48601	48679	4.78e-31
contig1	contig2	P	104	91.346	210151	210254	159602	159699	4.78e-31
contig1	contig2	P	95	92.632	255293	255384	67337	67431	6.18e-30
contig1	contig2	P	75	97.333	56640	56714	16836	16910	2.88e-28
contig1	contig2	F	76	97.368	239375	239449	31837	31912	2.88e-28
contig1	contig2	P	70	98.571	3521	3590	67305	67374	3.72e-27
contig1	contig2	P	69	98.551	3521	3589	68212	68280	1.34e-26
contig1	contig2	P	79	93.671	156307	156385	239223	239301	1.73e-25
contig1	contig2	F	79	93.671	135074	135148	94269	94347	2.24e-24
contig1	contig2	P	98	88.776	130810	130907	97777	97868	2.24e-24
contig1	contig2	F	79	92.405	268639	268714	68329	68405	3.75e-22
contig1	contig2	P	72	93.056	132878	132949	121790	121861	1.35e-21
contig1	contig2	P	66	95.455	239375	239440	153431	153495	4.85e-21
contig1	contig2	P	57	98.246	258355	258411	27328	27384	6.27e-20
contig1	contig2	F	71	92.958	233929	233997	133758	133827	6.27e-20
contig1	contig2	F	53	100	22453	22505	182298	182350	2.26e-19

contig1	contig2	F	71	91.549	198230	198300	240487	240555	8.11e-19
contig1	contig2	F	57	96.491	110108	110164	7319	7375	2.92e-18
contig1	contig2	F	69	91.304	181106	181172	142626	142693	3.77e-17
contig1	contig2	P	51	98.039	26490	26540	68668	68718	1.36e-16
contig1	contig2	P	192	75	14286	14474	30729	30919	1.76e-15
contig1	contig2	P	64	92.188	239417	239479	31660	31719	1.76e-15
contig1	contig2	F	56	94.643	239389	239443	88890	88944	1.76e-15
contig1	contig2	P	52	96.154	59817	59868	219341	219392	1.76e-15
contig1	contig2	P	54	94.444	130951	131004	28201	28254	6.32e-15
contig1	contig2	P	54	94.444	188995	189048	116314	116367	6.32e-15
contig1	contig2	F	49	97.959	268490	268538	144374	144421	6.32e-15
contig1	contig2	F	92	82.609	63108	63199	242025	242116	2.27e-14
contig1	contig2	P	43	100	122692	122734	51761	51803	8.17e-14
contig1	contig2	P	47	97.872	267033	267079	160291	160336	8.17e-14
contig1	contig2	F	47	97.872	190931	190976	200030	200076	8.17e-14
contig1	contig2	P	52	94.231	171736	171786	68313	68364	2.94e-13
contig1	contig2	P	50	94	142765	142814	48783	48832	1.06e-12
contig1	contig2	P	43	97.674	234942	234984	87765	87807	3.80e-12
contig1	contig2	F	49	93.878	181186	181234	121788	121836	3.80e-12
contig1	contig2	F	40	100	133004	133043	121799	121838	3.80e-12
contig1	contig2	P	43	97.674	171718	171760	142608	142650	3.80e-12
contig1	contig2	P	91	81.319	247825	247915	193826	193916	3.80e-12
contig1	contig2	P	91	81.319	76143	76233	193826	193916	3.80e-12

contig1	contig2	P	43	97.674	171718	171760	200828	200870	3.80e-12
contig1	contig2	P	44	97.727	234942	234985	200892	200934	3.80e-12
contig1	contig2	P	49	93.878	63089	63137	218090	218138	3.80e-12
contig1	contig2	F	57	89.474	267002	267058	7318	7373	4.92e-11
contig1	contig2	F	48	93.75	137226	137272	67418	67465	4.92e-11
contig1	contig2	F	38	100	164244	164281	197394	197431	4.92e-11
contig1	contig2	F	38	100	181106	181143	200846	200883	4.92e-11
contig1	contig2	P	97	79.381	13711	13807	31422	31518	1.77e-10
contig1	contig2	P	42	95.238	198461	198502	50842	50883	6.36e-10
contig1	contig2	F	55	89.091	268295	268349	68277	68329	6.36e-10
contig1	contig2	F	88	81.818	267571	267651	70667	70754	6.36e-10
contig1	contig2	F	35	100	3461	3495	28527	28561	2.29e-09
contig1	contig2	P	134	76.866	29384	29511	38972	39100	2.29e-09
contig1	contig2	F	54	88.889	87478	87530	64843	64896	2.29e-09
contig1	contig2	F	45	93.333	164207	164251	88904	88947	2.29e-09
contig1	contig2	P	38	97.368	191354	191391	111965	112002	2.29e-09
contig1	contig2	P	69	84.058	62945	63012	193901	193968	2.29e-09
contig1	contig2	P	41	95.122	57925	57965	219341	219381	2.29e-09
contig1	contig2	F	60	86.667	137177	137236	28417	28472	8.23e-09
contig1	contig2	P	34	100	178332	178365	31343	31376	8.23e-09
contig1	contig2	F	44	93.182	254499	254541	50744	50787	8.23e-09
contig1	contig2	F	34	100	14112	14145	160324	160357	8.23e-09
contig1	contig2	F	34	100	268333	268366	197263	197296	8.23e-09
contig1	contig2	P	40	95	179725	179764	228829	228868	8.23e-09

contig1	contig2	P	70	82.857	13461	13529	32040	32108	2.96e-08
contig1	contig2	F	33	100	235859	235891	40357	40389	2.96e-08
contig1	contig2	P	36	97.222	133000	133035	68279	68314	2.96e-08
contig1	contig2	F	39	94.872	132806	132844	87273	87311	2.96e-08
contig1	contig2	F	42	92.857	212465	212506	92913	92954	2.96e-08
contig1	contig2	F	39	94.872	141545	141583	93358	93396	2.96e-08
contig1	contig2	P	48	89.583	38453	38500	102081	102128	2.96e-08
contig1	contig2	F	33	100	268235	268267	149761	149793	2.96e-08
contig1	contig2	F	49	89.796	110108	110155	200000	200048	2.96e-08
contig1	contig2	F	54	87.037	87478	87530	27058	27111	1.06e-07
contig1	contig2	F	35	97.143	235001	235035	30159	30193	1.06e-07
contig1	contig2	F	41	92.683	179724	179764	51593	51633	1.06e-07
contig1	contig2	P	35	97.143	93114	93148	148612	148646	1.06e-07
contig1	contig2	P	39	94.872	38866	38904	149763	149800	1.06e-07
contig1	contig2	F	51	88.235	268515	268565	200880	200929	1.06e-07
contig1	contig2	P	32	100	268611	268642	210914	210945	1.06e-07
contig1	contig2	P	35	97.143	256762	256796	240082	240116	1.06e-07
contig1	contig2	P	69	84.058	251307	251373	245438	245501	1.06e-07
contig1	contig2	P	69	84.058	79625	79691	245438	245501	1.06e-07
contig1	contig2	P	35	97.143	101712	101746	248847	248881	1.06e-07
contig1	contig2	F	37	94.595	110185	110221	29809	29845	3.83e-07
contig1	contig2	P	47	89.362	137186	137231	31866	31911	3.83e-07
contig1	contig2	P	31	100	268237	268267	75626	75656	3.83e-07

contig1	contig2	P	38	94.737	269897	269933	132751	132788	3.83e-07
contig1	contig2	F	31	100	88506	88536	144373	144403	3.83e-07
contig1	contig2	F	31	100	169497	169527	152165	152195	3.83e-07
contig1	contig2	P	39	92.308	236194	236232	42341	42379	1.38e-06
contig1	contig2	P	40	92.5	38716	38754	65928	65967	1.38e-06
contig1	contig2	F	40	92.5	196224	196261	70414	70453	1.38e-06
contig1	contig2	F	33	96.97	173911	173943	75549	75581	1.38e-06
contig1	contig2	F	36	94.444	38869	38904	75621	75656	1.38e-06
contig1	contig2	F	41	92.683	271175	271214	101225	101263	1.38e-06
contig1	contig2	P	36	94.444	191386	191421	114190	114225	1.38e-06
contig1	contig2	F	46	89.13	178351	178396	142732	142775	1.38e-06
contig1	contig2	F	39	92.308	173981	174019	197347	197385	1.38e-06
contig1	contig2	P	49	87.755	268275	268323	200888	200934	1.38e-06
contig1	contig2	F	39	92.308	184456	184494	234905	234943	1.38e-06
contig1	contig2	P	35	94.286	196333	196367	28531	28565	4.95e-06
contig1	contig2	P	33	96.97	139025	139056	69801	69833	4.95e-06
contig1	contig2	P	41	90.244	135427	135467	87274	87314	4.95e-06
contig1	contig2	P	32	96.875	132973	133004	88916	88947	4.95e-06
contig1	contig2	F	41	90.244	52681	52721	100383	100423	4.95e-06
contig1	contig2	F	32	96.875	179733	179764	148132	148163	4.95e-06
contig1	contig2	F	29	100	43176	43204	204335	204363	4.95e-06
contig1	contig2	P	29	100	261140	261168	240178	240206	4.95e-06
contig2	contig2	P	2149	100	204689	206837	83399	85547	0

contig2	contig2	F	227	99.559	125881	126107	70183	70409	1.80e-114
contig2	contig2	F	238	81.513	175102	175328	63908	64138	7.16e-44
contig2	contig2	P	97	96.907	227326	227422	7476	7572	7.22e-39
contig2	contig2	P	103	95.146	7476	7578	227320	227422	7.22e-39
contig2	contig2	F	85	100	200790	200874	142570	142654	3.36e-37
contig2	contig2	F	89	97.753	64839	64927	27054	27142	4.34e-36
contig2	contig2	P	122	90.164	105064	105185	47240	47356	4.34e-36
contig2	contig2	F	75	100	93617	93691	72210	72284	1.22e-31
contig2	contig2	F	119	87.395	114002	114119	113703	113820	1.57e-30
contig2	contig2	F	72	100	68212	68283	67306	67377	5.66e-30
contig2	contig2	F	88	93.182	32252	32339	7832	7919	7.32e-29
contig2	contig2	F	100	90	114229	114328	111350	111449	7.32e-29
contig2	contig2	F	71	97.183	200893	200962	87765	87835	1.58e-25
contig2	contig2	P	67	95.522	204506	204572	101467	101532	1.23e-21
contig2	contig2	P	69	94.203	153429	153495	31837	31905	4.44e-21
contig2	contig2	F	65	95.385	164811	164875	47389	47453	4.44e-21
contig2	contig2	P	72	93.056	31837	31908	153426	153495	4.44e-21
contig2	contig2	P	69	92.754	150395	150463	71166	71233	2.06e-19
contig2	contig2	F	53	100	200644	200696	87668	87720	2.06e-19
contig2	contig2	P	102	83.333	155702	155803	12363	12464	2.67e-18
contig2	contig2	P	105	82.857	12363	12467	155699	155803	2.67e-18
contig2	contig2	P	61	93.443	229739	229799	27278	27338	3.45e-17
contig2	contig2	F	51	98.039	68668	68718	48628	48678	1.24e-16
contig2	contig2	F	104	82.692	198026	198129	32295	32392	4.47e-16
contig2	contig2	P	48	97.917	20068	20073	14726	14731	5.78e-15

					4	1	4	1		
contig2	contig2	F	53	94.34	88890	88941	31852	31903	7.48e-14
contig2	contig2	P	56	92.857	150239	150292	71239	71294	7.48e-14
contig2	contig2	F	53	94.34	213249	213300	73008	73060	7.48e-14
contig2	contig2	P	59	91.525	248578	248636	107067	107124	7.48e-14
contig2	contig2	P	47	97.872	200030	200076	160291	160336	7.48e-14
contig2	contig2	F	42	100	124152	124193	87651	87692	2.69e-13
contig2	contig2	F	47	95.745	32411	32457	7961	8007	9.67e-13
contig2	contig2	P	95	81.053	156188	156280	11928	12020	1.25e-11
contig2	contig2	P	53	92.453	153431	153481	88890	88941	1.25e-11
contig2	contig2	P	46	95.652	229833	229878	98260	98304	1.25e-11
contig2	contig2	F	57	89.474	199999	200055	7318	7373	4.50e-11
contig2	contig2	F	78	83.333	198026	198103	7875	7950	4.50e-11
contig2	contig2	P	47	93.617	149759	149805	75614	75660	4.50e-11
contig2	contig2	F	47	93.617	235819	235865	152370	152416	4.50e-11
contig2	contig2	P	40	97.5	228829	228868	51594	51633	1.62e-10
contig2	contig2	F	40	97.5	234713	234752	234674	234713	1.62e-10
contig2	contig2	F	66	86.364	200865	200925	147293	147357	5.82e-10
contig2	contig2	P	158	75.316	226117	226267	219224	219379	5.82e-10
contig2	contig2	F	42	95.238	240210	240251	7846	7886	2.09e-09
contig2	contig2	F	42	95.238	240210	240251	32266	32306	2.09e-09
contig2	contig2	P	35	100	2517	2551	195256	195290	2.09e-09
contig2	contig2	F	35	100	229915	229949	214382	214416	2.09e-09
contig2	contig2	F	47	91.489	21302	21307	28544	28589	7.53e-09

					4	0			
contig2	contig2	F	33	100	51075	51107	51018	51050	2.71e-08
contig2	contig2	P	70	82.857	87242	87310	87242	87310	2.71e-08
contig2	contig2	P	36	97.222	228825	228860	148132	148167	2.71e-08
contig2	contig2	P	50	88	216650	216699	21194	21243	9.74e-08
contig2	contig2	F	57	85.965	165090	165144	39356	39412	9.74e-08
contig2	contig2	F	35	97.143	248847	248881	41096	41130	9.74e-08
contig2	contig2	F	38	94.737	148132	148169	51602	51639	9.74e-08
contig2	contig2	F	50	88	55942	55991	55371	55420	9.74e-08
contig2	contig2	F	35	97.143	212557	212591	82995	83029	9.74e-08
contig2	contig2	F	61	85.246	114165	114225	113928	113985	9.74e-08
contig2	contig2	F	57	85.965	200403	200459	128903	128958	9.74e-08
contig2	contig2	P	59	86.441	142662	142719	31788	31840	3.50e-07
contig2	contig2	F	31	100	137408	137438	87784	87814	3.50e-07
contig2	contig2	P	34	97.059	232284	232317	122669	122702	3.50e-07
contig2	contig2	P	31	100	122672	122702	232284	232314	3.50e-07
contig2	contig2	F	30	100	214491	214520	47958	47987	1.26e-06
contig2	contig2	P	33	96.97	67174	67206	65935	65967	1.26e-06
contig2	contig2	P	30	100	142638	142667	68308	68337	1.26e-06
contig2	contig2	F	34	97.059	201046	201078	71096	71129	1.26e-06
contig2	contig2	F	30	100	92403	92432	88882	88911	1.26e-06
contig2	contig2	F	30	100	192608	192637	144410	144439	1.26e-06
contig2	contig2	P	35	94.286	105019	105053	37376	37410	4.53e-06
contig2	contig2	P	32	96.875	121799	121830	68279	68310	4.53e-06
contig2	contig2	P	29	100	240219	240247	198007	198035	4.53e-06

contig2	contig1	P	363	79.339	28855	29214	15648	16003	6.97e-64
contig2	contig1	P	442	76.244	29498	29920	14923	15348	1.53e-50
contig2	contig1	P	313	79.233	41095	41396	101448	101747	7.11e-49
contig2	contig1	P	75	100	85541	85615	110063	110137	1.22e-31
contig2	contig1	P	78	96.154	16836	16913	56637	56714	2.63e-28
contig2	contig1	P	210	74.286	30729	30937	14268	14474	1.61e-15
contig2	contig1	P	51	96.078	28204	28254	130951	131001	5.78e-15
contig2	contig1	P	57	92.982	116314	116370	188992	189048	5.78e-15
contig2	contig1	P	40	100	87768	87807	234942	234981	3.48e-12
contig2	contig1	P	131	77.099	38975	39100	29384	29508	2.09e-09
contig2	contig1	P	41	95.122	111965	112005	191351	191391	2.09e-09
contig2	contig1	P	43	93.023	228829	228871	179722	179764	7.53e-09
contig2	contig1	P	76	81.579	32040	32114	13455	13529	2.71e-08
contig2	contig1	P	35	97.143	132754	132788	269897	269930	3.50e-07

P: Palindromic repeat; F: forward repeat.

Table S7. Gene transfer between chloroplast and mt genomes in *P. arguta.*

No	Identity%	Length	Mismatches	Gap	Cp start	Cp end	Mt start	Mt end	Gene(cp)	Gene(mt)
1	99.854	13000	16	3	47239	60237	149169	162166	rps4(partical:84.98%);trnT-UGU;trnL-UAA;trnF-GAA;ndhJ;ndhK;ndhC;trnV-UAC;trnM-CAU;atpE;atpB;rbcL;accD(partical:60.12%)	trnT-TGT;trnF-GAA;trnM-CAT
2	99.956	9193	3	1	133184	142376	17523	8332	trnR-ACG;rrn5;rrn4.5;rrn23;trnA-UGC;trnI-GAU;rrn16;trnV-GAC	trnV-GAC;trnI-GAT;trnA-TGC;trnR-ACG
3	99.956	9193	3	1	102262	111454	8332	17523	trnV-GAC;rrn16;trnI-GAU;trnA-UGC;rrn23;rrn4.5;rrn5;trnR-ACG	trnV-GAC;trnI-GAT;trnA-TGC;trnR-ACG
4	99.906	2118	2	0	42167	44284	2370	253	psaA(partical:48.60%);ycf3(partical:15.49%)	
5	87.798	1721	144	30	83219	84934	106390	104731	rpl14;rpl16(partical:77.93%)	
6	73.536	888	182	39	103538	104401	236017	235159	rrn16(partical:57.99%)	rrn18(partical:44.39%)
7	73.53	888	182	39	140237	141100	235159	236017	rrn16(part	rrn18(part

	6								ical:57.99%)	ical:44.39%)
8	96.429	84	3	0	133075	133158	171315	171232	trnN-GUU	trnN-GTT
9	96.429	84	3	0	111480	111563	171232	171315	trnN-GUU	trnN-GTT
10	96.104	77	3	0	137138	137214	190691	190615	rrn23(partical:2.74%)	
11	96.104	77	3	0	107424	107500	190615	190691	rrn23(partical:2.74%)	
12	97.297	74	2	0	142692	142765	190750	190677		
13	97.297	74	2	0	101873	101946	190677	190750		
14	100	34	0	0	108043	108076	190943	190910	rrn23(partical:1.21%)	
15	100	34	0	0	136562	136595	190910	190943	rrn23(partical:1.21%)	
16	100	30	0	0	155365	155394	118831	118802	ycf2(partical:0.43%)	
17	100	30	0	0	89244	89273	118802	118831	ycf2(partical:0.43%)	
18	96.875	32	1	0	114392	114423	214926	214895	ndhF(partical:1.40%)	
19	85.251	1695	154	44	66331	67958	139834	141499	psbJ;psbL;psbF;psbE	
20	83.934	971	83	31	30741	31679	135134	134205	psbM	
21	85.038	528	43	15	68818	69336	141859	142359	trnW-CCA;trnP-UGG	trnW-CCA;trnP-TGG
22	87.075	294	34	4	68371	68662	141577	141868	petL	
23	89.143	175	9	5	29899	30072	135292	135127	petN	
24	93.65	126	8	0	31668	31793	134160	134035	trnD-GU	trnD-GT

	1								C	C
25	89.796	147	7	1	36268	36414	235708	235570	psbC(partical:10.34%)	
26	97.531	81	2	0	14	94	149379	149459	trnH-GUG	trnH-GTG
27	93.671	79	5	0	54378	54456	239301	239223	trnM-CAU	trnM-CAT
28	97.368	38	1	0	147617	147654	137200	137163		
29	97.368	38	1	0	96984	97021	137163	137200		
30	100	34	0	0	136562	136595	160357	160324	rrn23(partical:1.21%)	
31	100	34	0	0	108043	108076	160324	160357	rrn23(partical:1.21%)	

Table S8. Pi of PCGs in *P. arguta* mt genome.

No.	Region	Pi	Total Number of mutations	Length
1	*atp1*	0.0678	253	1536
2	*atp4*	0.07739	122	600
3	*atp6*	0.04718	104	1496
4	*atp8*	0.06939	115	495
5	*atp9*	0.10852	68	282
6	*ccmB*	0.04817	108	621
7	*ccmC*	0.05538	124	834
8	*ccmFc*	0.05716	268	1446
9	*ccmFn*	0.04436	276	1845
10	*cob*	0.03647	143	1206
11	*cox1*	0.03702	162	1587
12	*cox2*	0.05887	129	1434
13	*cox3*	0.03182	83	798
14	*matR*	0.04955	317	2041
15	*mttB*	0.07826	77	864
16	*nad1*	0.02811	62	979
17	*nad2*	0.02717	122	1467
18	*nad3*	0.05291	45	357
19	*nad4*	0.04294	165	1488
20	*nad4L*	0.02442	17	303
21	*nad5*	0.03172	160	2124
22	*nad6*	0.03524	77	661
23	*nad7*	0.02399	82	1185
24	*nad9*	0.02487	48	573
25	*rpl10*	0.05168	52	507
26	*rpl2*	0.03072	57	1026
27	*rpl5*	0.03634	52	564
28	*rps1*	0.05504	56	609
29	*rps12*	0.04174	50	378
30	*rps13*	0.05752	55	351
31	*rps14*	0.01782	13	303
32	*rps19*	0.04561	22	291
33	*rps3*	0.06964	348	1794
34	*rps4*	0.07876	165	1143
35	*rps7*	0.0563	49	447
36	*rrn18*	0.04983	245	2129
37	*rrn26*	0.03318	317	3753
38	*rrn5*	0.03003	13	122
39	*sdh3*	0.04686	31	384
40	*sdh4*	0.02773	26	474
